# Association between Eosinophil Count and Cortisol Concentrations in Equids Admitted in the Emergency Unit with Abdominal Pain

**DOI:** 10.3390/ani14010164

**Published:** 2024-01-04

**Authors:** María Villalba-Orero, María Dolores Contreras-Aguilar, Jose Joaquín Cerón, Beatriz Fuentes-Romero, Marta Valero-González, María Martín-Cuervo

**Affiliations:** 1 Hospital Clínico Veterinario Complutense, Departamento de Medicina y Cirugía Animal, Facultad de Veterinaria, Universidad Complutense de Madrid, 28040 Madrid, Spain; mvorero@ucm.es; 2 Interdisciplinary Laboratory of Clinical Analysis of the University of Murcia (Interlab-UMU), Department of Animal Medicine and Surgery, Veterinary School, Regional Campus of International Excellence Mare Nostrum, Campus de Espinardo, University of Murcia, 30100 Murcia, Spain; jjceron@um.es; 3 Veterinary Teaching Hospital, University of Extremadura, Avda de la Universidad s/n, 10005 Cáceres, Spain; beatriz.fro@gmail.com (B.F.-R.); martavalero94@hotmail.es (M.V.-G.); 4 Grupo MECIAN, Departamento de Medicina Animal, Facultad de Veterinaria, Campus de Cáceres, Universidad de Extremadura, 10004 Cáceres, Spain; mariamc@unex.es

**Keywords:** eosinophils, cortisol, horses, survival, biomarker, colic

## Abstract

**Simple Summary:**

Acute stress can manifest physiologically through a “stress leukogram” (neutrophilia, eosinopenia, lymphopenia, and mild monocytosis), with eosinopenia as one of its main indicators. Changes in cortisol concentrations, the primary stress biomarker, whether measured in blood or saliva, strongly correlate with the severity of gastrointestinal diseases in equids. Recently, eosinophil count has been used to identify critically ill horses and as a prognostic marker in horses with Systemic Inflammatory Response Syndrome or SIRS. However, the relationship between both parameters has not been proven. Therefore, the objective of this study was to assess the possible differences in eosinophil count in horses with acute abdominal diseases that survived and non-survived upon hospitalization and its relationship with salivary cortisol. It was demonstrated that non-surviving horses showed the lowest eosinophil count, and a strong correlation (negative) was observed between cortisol and eosinophil count. Thus, eosinophil count could be a potential prognosis biomarker in horses with acute abdominal diseases.

**Abstract:**

Stress leukogram includes eosinopenia as one of its main markers (neutrophilia, eosinopenia, lymphopenia, and mild monocytosis). Cortisol is the main stress biomarker, which is also strongly correlated with the severity of gastrointestinal diseases. This study aimed to determine the relationship between salivary cortisol and the eosinophil cell count (EC) in equids with abdominal pain. To do this, 39 horses with abdominal pain referred to an emergency service were included. All samples were taken on admission, and several parameters and clinical data were included. Equids were classified according to the outcome as survivors and non-survivors. Non-surviving equids presented higher salivary cortisol concentrations (Non-Survivors: 1.580 ± 0.816 µg/dL; Survivors 0.988 ± 0.653 µg/dL; *p* < 0.05) and lower EC (Non-Survivors: 0.0000 × 10^3^/µL (0.000/0.0075); Survivors: 0.0450 × 10^3^/µL (0.010/0.1825); *p* < 0.01). In addition, the relationship between salivary cortisol concentration, EC, and the WBC was determined. Only a strong correlation (negative) was observed between cortisol and EC (r = −0.523, *p* < 0.01). Since cortisol is not an analyte that can be measured routinely in clinical settings such as emergencies, the EC could be a good alternative. While the results are promising, further studies are needed before EC can be used confidently in routine practice to predict survival in cases of abdominal pain.

## 1. Introduction

Acute abdominal pain due to gastrointestinal tract injury in horses is a frequent emergency in equine medicine with a reserved to poor prognosis [1,2]. Intensive care treatment in these horses can be expensive; thus, establishing a rapid and accurate clinical prognosis in horses with colic is essential. However, the time required for diagnosis and treatment is limited, especially when surgical intervention is indicated [1], and in some cases, a quick decision to treat or humanely euthanize the patient is a challenge. 

Cortisol is the main stress biomarker, which is also strongly correlated with the severity of gastrointestinal disease in horses [2,3]. In horses with colic, cortisol measurement is a very useful marker of illness and severity, as acute abdominal pain leads to sympathetic nervous system activation [1,4]. Although there is limited information on the physiopathological mechanisms of cortisol release in adult horses with colic, cases of adrenal gland lesions have been described in horses with gastrointestinal ischemic pathologies during postmortem examination [2], which would partially account for the endocrine alterations found in these patients. Horses present high blood cortisol concentrations [1], and those with a marked risk of death show the highest concentrations of cortisol [4]. The measurement of salivary cortisol concentrations in horses has been previously validated, demonstrating its reliability as a non-invasive method for assessing stress levels [5,6]. Salivary cortisol measurements in equine clinical practice have provided valuable insights into horses’ physiological responses to various stressors [7]. Additionally, a very strong to moderate correlation has been observed between salivary and plasma cortisol concentrations, allowing for reliable use in either type of sample [8,9]. Thus, salivary cortisol can be considered a reliable and robust substitute biomarker for blood cortisol in horses for its welfare evaluation since saliva collection is a pain-free procedure and a non-invasive sample collection technique. For this reason, salivary cortisol has been extensively studied in many species to try to understand the neuroendocrine mechanisms underlying stress. Understanding its effects can aid in obtaining more precise diagnoses and targeted therapeutic interventions [10,11]. Despite the valuable usefulness of cortisol in reaching a prognosis, cortisol still cannot be easily measured in routine clinical conditions, especially in emergencies, and it is unavailable in many equine clinics. The equipment that allows its quantification is usually located in large hospitals or reference laboratories, making immediate measurement challenging. In addition, to the author’s knowledge, no point-of-care (POC) automatic analyzers are currently available to measure cortisol in horses in field conditions. Thus, finding other analytes related to the degree of stress that are inexpensive and easily available may enable clinicians to achieve a quick prognosis in horses with colic admitted to hospitals. 

White blood cell count (WBC) could indicate the severity of gastrointestinal disease; however, horses with acute abdominal pain may develop leucopenia, which can be hidden based on the stress leucogram, making it virtually impossible to establish a real relationship between cortisol and WBC count [3]. Eosinopenia has become an attractive biomarker in humans, offering a valuable tool for prognosis in critical conditions. It represents a marker of acute inflammation [12], infection [13], sepsis [14,15], and mortality [16]. Recently, eosinophil cell count (EC) has also been pointed out as a valuable parameter for poor prognosis in critically ill horses [3]. In this report, horses admitted to the intensive unit with Systemic Inflammatory Response Syndrome (SIRS) showed lower eosinophil counts compared to healthy ones, and non-surviving animals presented the lowest EC. Systemic Inflammatory Response Syndrome is a complex physiological response observed in horses experiencing acute abdominal pain and is often associated with severe gastrointestinal issues [2]. This syndrome triggers a cascade of inflammatory reactions throughout the body and can be caused by various stimuli such as infection, trauma, or ischemia. In the context of equine veterinary medicine, SIRS becomes particularly relevant when addressing acute abdominal pain, which can be indicative of serious conditions like colic. SIRS is defined by a set of clinical and laboratory criteria indicating a systemic inflammatory condition. In horses, these criteria include abnormal heart rate, increased respiratory rate, abnormal temperature, changes in white blood cell count, and other signs of systemic distress. When these parameters exceed the established thresholds, it signals an uncontrolled inflammatory response that can lead to further complications. Furthermore, when horses experience acute abdominal pain, the underlying pathology can trigger a systemic inflammatory response. This response is the body’s attempt to mitigate the damage, fight infection, and restore homeostasis. However, the inflammatory response can become dysregulated in severe colic cases, leading to SIRS [3].

As the inflammation cascade builds up, chemotactic molecules released from inflammatory cells during the inflammatory response lead to the sequestration of circulating eosinophils at the site of inflammation, thus decreasing the number of circulating eosinophils [17]. Indeed, an EC decrease is part of the canonical stress leukogram (neutrophilia, eosinopenia, lymphopenia, and mild monocytosis) in response to cortisol release. The cortisol influence in the eosinophil kinetics was evaluated by Andersen et al. [18] in an experimental test performed in rats, where it was demonstrated that steroids cause a reversible sequestration of blood eosinophils, and the long-term steroid influence may delay the release of mature eosinophils from the bone marrow into the blood stream. Acute abdominal pain in horses is a syndrome often characterized by acute inflammation and different degrees of infection, septicemia, and/or sepsis [19,20]. In addition, salivary cortisol has been demonstrated to increase in horses suffering acute abdominal disease [11]. However, since cortisol in horses is not a parameter that can be measured routinely in clinical settings such as emergencies, the EC may be a good complement or even an alternative as a prognostic marker. However, the relationship between both parameters has not been proven. Therefore, this study aims (1) to evaluate the usefulness of eosinopenia as a predictor of non-survival in equids with acute abdominal pain and (2) to determine the relationship between eosinophil counts and concentrations of the stress marker cortisol. 

## 2. Materials and Methods

### 2.1. Animals and Study Design

All owners approved the usefulness of the clinical data with informed consent.

This observational study did not require any deviation from routine medical practice. A convenience sampling approach was adopted, and privately owned equids with abdominal pain received in the emergency service between 2019 and 2020 at the Veterinary Teaching Hospital of the University of Extremadura were included. All equids were submitted by referring veterinarians and assessed upon arrival by the specialist veterinarian or resident on duty. Equids were classified according to the survival outcome as survivors and non-survivors. An equid was considered a non-survivor if it died or was euthanized due to the severity of its pathology. Equids euthanized for economic reasons were not included in the study. All equids were diagnosed based on treatment response and conducted tests (medical colics), findings during surgery, or postmortem examination. Only equids over two years old were included since horses under one year may exhibit normal values for some hematological and biochemical parameters different from adult horses, and the stress response differs in very young animals, which could introduce bias into the results.

### 2.2. Sample Collection and Measurements

Saliva samples were obtained on admission as previously reported [21] by soaking 5 × 2 × 2 cm^3^ polypropylene sponges (Esponja Marina, La Griega E. Koronis, Madrid, Spain) with saliva, clipping them to an independent flexible thin metal rod and introduced into the horses’ mouth vestibule across the third or fourth maxillary premolar for 1 min. Then, the sponges were placed in a commercially available device (Salivette, Sarstedt, Aktiengesellschaft & Co., Nümbrecht, Germany) immediately after sampling. Salivary samples from equids which were referred to the hospital within less than 12 h from the onset of clinical signs and, therefore, fasted less than that period were obtained after the equids’ mouth was washed using a manual suction pump usually employed in nasogastric intubation (Maxi Drencher 300 mL with feeding cannula 20 cm, ASTRO S.r.l., Reggio Emilia RE, Italy), and saliva was retrieved 5 min after washing. These conditions guaranteed a salivary dirtiness degree ≤1 according to a color scale previously reported (0–4 score) [21]. Only two horses were referred to the hospital after 12 h of receiving any feed after an ambulatory treatment; thus, their mouths were not washed. Then, salivary devices were centrifuged at 3000× *g* for 10 min at 4 °C to obtain saliva specimens, which were stored at −80 °C until analysis (less than six months). Blood samples were taken after saliva samples by venipuncture of the jugular vein and were collected in microtubes containing ethylenediaminetetraacetic acid anticoagulant (EDTA) on admission.

Several descriptive and clinical parameters were obtained as part of the regular diagnosis protocol. From all of them, the demographic data extracted were breed, sex, and age. The clinical parameters included were rectal temperature, heart rate (HR), respiratory rate (RR), mucous membrane appearance, and white blood cell count (WBC).

The WBC was performed by a semiautomatic electronic blood cell counter (Sysmex F-800). Moreover, the EC was performed by manual Diff-Quick staining by classifying 200 WBCs on a blood smear to determine the percentage of each type of WBC present. The percentage of eosinophils was multiplied by the total WBC × 10^3^ /µL to obtain the absolute count of this WBC type [22,23]. The leukocyte differential count was performed manually to detect significant toxic neutrophil changes. Salivary cortisol (µg/dL) was measured with an immunoassay system (Immulite 1000, Siemens Healthcare Diagnostic, Forchheim, Germany) using a solid-phase competitive enzyme-amplified chemiluminescent immunoassay. The assay has been previously validated in horse saliva [24], showing an intra- and inter-assay imprecision lower than 15% and linearity after serial sample dilution.

### 2.3. Data Analysis

Normality distribution was assessed through visual inspection of normal curves in histograms (symmetric and middle-tailed profiles) and a Kolmogorov–Smirnov test. Data were presented as mean ± standard deviations (SD) for variables with a normal distribution and as medians with interquartile ranges (IQT; 25th/75th) when normality could not be assumed. Firstly, the data were grouped into survival and non-survival, and a Student’s T-test or Mann–Whitney test (parametric or non-parametric, respectively) was applied to determine differences in cortisol concentrations, EC, and WBC. Pearson’s or Spearman’s pairwise correlations (parametric or non-parametric, respectively) were used to investigate the relationship between salivary cortisol, EC, and WBC. Correlations between the variables were interpreted as follows: r < 0.19—no correlation, r > 0.20 and <0.29—weak correlation, r > 0.3 and <0.49—moderate correlation, r > 0.5 and <0.69—strong correlation, r > 0.7—very strong correlation [25]. All statistical analyses were performed using Prism version 9 (San Diego, CA, USA).

## 3. Results

### 3.1. Demographic and Clinical Variables

A total of 39 equids, with a median age of 8.0 years (5/10), were included in the study. All the study data are provided in Supplementary Table S1. Among them, 53.8% were males, 30.8% were females, and 15.4% were geldings; 33.3% were Spanish Pure Breed, 25.6% were crossbred, 15.4% were hot-blooded, 10.3% were warm-blood, and 7.7% were Lusitano, while ponies and donkeys represented 2.6% each one (breed data were unavailable for 2.6% of the 39 equids). At admission, the horses presented a rectal temperature of 37.8 ± 0.7 °C, HR of 59 ± 22 ppm, and RR of 16 (13/24) bpm. The mucous membrane color was pink in 51.3%, congested in 30.8%, pale in 10.3%, and cyanotic in 2.6% of the equids (mucous membrane color was not registered for 5% of 39 equids).

The location of the abdominal damage was 51.3% in the large intestine, 30.8% in the small intestine, 7.7% in both, 5.1% in the stomach, and 2.6% in the uterus. The lesion was non-strangulated in 84.6% of the equids, whereas strangulation or a rupture was observed in 15.4% of the cases. Total survivors were 76.9%, and non-survivors were 20.5%.

The demographic and clinical data of survivors and non-survivors are shown in Table 1. Most non-surviving horses were males and geldings (80%), aged 6.5 (5/9.8). At admission, these horses presented a rectal temperature of 38.5 ± 0.5 °C, HR of 92 ± 17 ppm, RR of 40 (24/43) bpm, and congestive mucous membrane in the majority (75.0%). In non-surviving horses, the large intestine was damaged in 50.0% of the cases, and the lesions, regardless of localization, were due to strangulation or rupture in 50% of the cases.

### 3.2. Salivary and Blood Analyses

**Figure 1:**
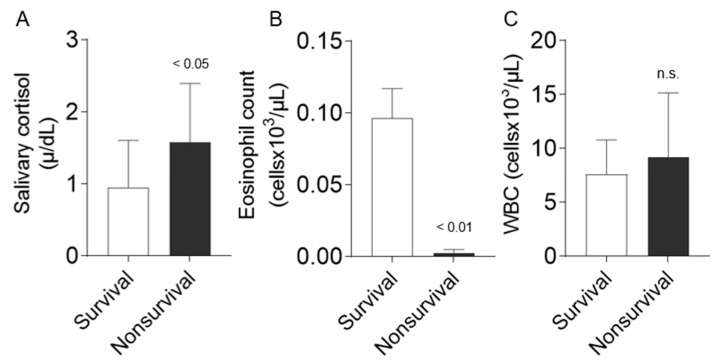
Comparison between survival and non-survival equids regarding salivary cortisol (*n* = 39, **A**), EC (n = 30, **B**), and WBC (n = 35, **C**) in horses admitted to an equine hospital with acute abdominal pain. Data are presented as mean ± SD (**A**,**C**) and median ± standard error of the mean (**B**). Statistical analysis was performed using a Student’s *t*-test if data showed normal distribution or the Mann–Whitney test if no normal distribution was presented. n.s. no significance.

Salivary cortisol was significantly higher in non-surviving equids compared to the surviving patients (1.580 ± 0.816 µg/dL vs. 0.988 ± 0.653 µg/dL; *p* = 0.03; Figure 1A]). In addition, eosinophils showed marked lower counts in non-surviving patients compared to survivors (0.0000 × 10^3^/µL (0.000/0.0075) vs. 0.0450 × 10^3^/µL (0.010/0.1825); *p* = 0.001; Figure 1B), while no differences were found in total WBC between non-surviving and surviving (8695 ± 3704 × 10^3^/µL vs. 7350 ± 3396 × 10^3^/µL; n.s.; Figure 1C).

**Figure 2:**
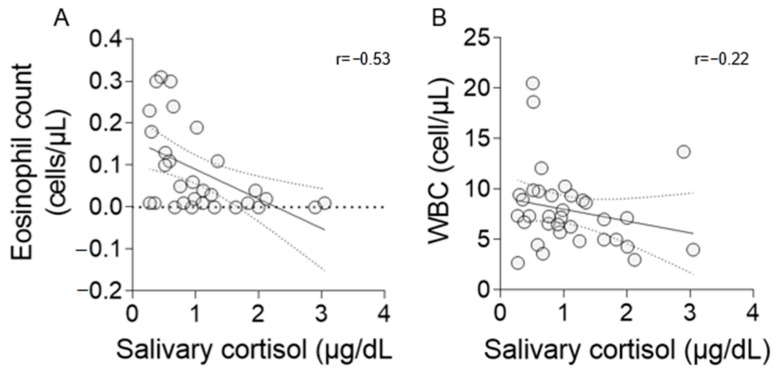
Correlation coefficient (r) between salivary cortisol, EC (**A**), and WBC (**B**). Statistical analysis was performed using Spearman’s rho coefficient (**A**) and Pearson’s coefficient (**B**). Graphics show a regression line with 95% confidence bands.

A strong negative correlation was found between salivary cortisol concentrations and EC (r = −0.53, *p* = 0.002; Figure 2A). However, a weak correlation was observed between cortisol and WBC (r = −0.22, n.s.; Figure 2B).

## 4. Discussion

Acute abdominal pain in horses is a critical condition, and early diagnosis plays a crucial role in improving prognosis and reducing mortality rates. Horses presenting with signs of SIRS or endotoxemia alongside acute abdominal pain tend to have a poorer prognosis compared to those without systemic involvement [1,20]. The identification of biomarkers for recognizing critically ill equine patients is essential to establishing accurate prognoses. In daily clinical practice, developing reliable biomarkers is imperative for timely intervention and effective management, ultimately enhancing survival outcomes for horses experiencing acute abdominal pain.

This study is the first to compare cortisol, which is strongly correlated with the severity of gastrointestinal disease in horses [2], with EC in equids admitted to an equine hospital with acute abdominal pain. Despite numerous studies published in human medicine regarding eosinopenia as a prognostic marker, there is scarce literature discussing its correlation with cortisol concentrations [26].

In our study, equids with acute abdominal pain showed a strong correlation between cortisol concentrations and eosinopenia. In addition, our results show that non-surviving patients present a lower eosinophil count compared to surviving patients. Eosinophils in the body are continuously regulated. On the one hand, the initial eosinopenia occurring in response to acute damage is due to a rapid peripheral sequestration of circulating eosinophils [12,27]. In addition, acute injury also triggers acute stress, which induces eosinopenia mediated by adrenal glucocorticosteroid and epinephrine release [12,14,16]. This eosinopenia in response to circulating sympathetic mediators may explain the relationship found in our study between these two stress parameters (eosinopenia and cortisol) and would support the usefulness of eosinophil count as a biomarker instead of cortisol.

In critically sick human patients, eosinopenia on admission has been reported to be associated with a worse prognosis during hospitalization; moreover, the deeper the eosinopenia, the poorer the prognoses [16,28,29,30]. Similar results have also been found for when the existing pathology affects the digestive tract and has also been observed in other systemic disorders [31,32,33,34]. Eosinophils are components of the innate immune system and exhibit the ability to respond to pathogen-related molecules. As a result, a significant reduction in peripheral blood EC occurs due to infection. This phenomenon has been associated with acute bacterial infections in human patients, playing a prominent role in clearing infections [5,35]. Eosinophil margination and recruitment to the sites of infection contribute to the acute decline in circulating eosinophils associated with acute bacterial infections [2]. Indeed, studies in humans also showed that the percentage of eosinophils in the peripheral blood smear decreased as the number of bacteria-positive blood cultures per patient increased [27,36]. Nevertheless, studies concerning EC in equine patients have focused on eosinophilia [37] but not on eosinopenia as a prognosis biomarker of disease. Eosinophilia in horses is related to allergic airway disease, gastrointestinal helminth infections, allergic skin disease, multisystemic eosinophilic epitheliotropic disease, eosinophilic disease confined to the intestine, phycomycosis, and neoplasia [37]. Recently, the prognosis of eosinopenia as a biomarker in critically ill horses was described for the first time [3]. According to this result and those shown in human patients with gastrointestinal diseases, we found that EC in equids with acute abdominal pain are also lower in non-surviving patients. It is likely that equids with severe intestinal damage present higher degree of inflammation, with or without infection, that results in eosinopenia. The relevance of this finding relies on the fact that EC is routinely determined on admission in most equine clinics, enhancing its utility.

Total WBC provides general information about immunity status and inflammation. However, WBC count in horses on admission is not a useful biomarker of prognosis and requires the assessment of the changes [3]. Based on these previous results, it was expected that no differences in the WBC count between surviving and non-surviving horses would be found in our study. In addition, equids presenting acute abdominal injury may show leucocytosis or leukopenia depending on many factors related to the underlying disease, making this a non-reliable biomarker to predict patients’ odds of dying.

Conversely, salivary or serum cortisol is one of the most reliable prognostic markers of survival outcomes across all species. In human medicine, a strong correlation has been observed between cortisol concentrations and mortality in patients with coronary disorders [38,39], severe systemic diseases [40], tumors [41], and a wide variety of disorders [42]. Furthermore, its usefulness has been demonstrated in assessing survival in horses with digestive disorders [43]. In equids, the vivid pain caused by gastrointestinal disorders is known to induce significant stress. This stress leads to the release of cortisol from the adrenal gland due to the stimulation of the hypothalamic-pituitary-adrenal axis [44]. Although studies on the mechanisms of cortisol release in horses are limited, there are several publications on serum cortisol concentrations in horses with colic pain [4,45]. Salivary cortisol has been widely used as a prognostic marker in both human and equine patients for various diseases [46,47,48]. Additionally, a good correlation has been observed between salivary cortisol and plasma cortisol [49,50]. Consistent with the previously mentioned reports, salivary cortisol in our study showed higher concentrations in the non-surviving equids compared to those that survived. However, although significative, these differences showed an important overlap among EC between both groups, suggesting that eosinopenia could be a better prognostic biomarker than high salivary cortisol concentrations in equid with colic pain. Nevertheless, this must be further demonstrated in other studies using a larger population.

Horses presenting with acute abdominal pain that exhibit clinical and laboratorial signs of SIRS or endotoxemia have a worse prognosis than those without systemic involvement. Within the definition of SIRS, leukopenia or leukocytosis is included as an inclusion criterion, highlighting the importance of hematology as a classical inflammation marker in all species. Furthermore, neutropenic leukopenia is the most evident component among the laboratorial parameters routinely evaluated that indicates endotoxemia. In addition, it has been experimentally demonstrated that neutropenia occurs immediately after endotoxin administration [51]. This fact highlights the importance of white blood cell count for identifying critically ill horses. Otherwise, regarding the relationship between WBC count and cortisol concentrations, the release of cortisol causes the so-called “stress leukogram” which is characterized by the presence of neutrophilia, lymphopenia, and eosinopenia, where cortisol can cause deeper changes in WBC counts than metanephrines [52]. However, neutropenia due to inflammation can mask the stress leukogram. This is where the importance of EC and its relationship with cortisol lies. Indeed, recently, the relationship between eosinopenia and SIRS in critically ill horses has been assessed, suggesting the existence of a connection between eosinopenia and high cortisol concentrations in these patients [3].

Our study presents several limitations. Firstly, it was an observational, prospective and single-center study. As a result, the relevance of the results could be limited in different centers or multicentric studies. Another limitation of this work is that the hours of the day and the time of year when the equids were referred to our hospital was not taken into account, and, therefore, the effects of the circadian rhythm of cortisol release throughout the day and at different seasons may have biased some of the results. Lastly, since EC is low in normal equids, caution must be taken when interpreting these results as a cut-off value cannot be obtained from our study.

## 5. Conclusions

This study highlights that non-surviving equids admitted to an equine hospital with colic present low eosinophil counts. The total number of eosinophils shows a negative correlation with salivary cortisol concentrations. Therefore, eosinopenia could be a reliable complement or even an alternative to cortisol measurements as a stress biomarker of prognosis in cases of equine colic. While the results are promising, further studies are needed before EC can be used confidently in routine practice to predict survival in cases of abdominal pain in equids.

## Figures and Tables

**Table 1:** Demographic and clinical variables of 76.9% survivor and 20.5% non-survivor horses admitted to an equine hospital with acute abdominal pain (N = 39).

	Survival		Non-Survival	
Parameter	Mean ± SD or Median (25th/75th)	*n* (%)	Mean ± SD or Median (25th/75th)	*n* (%)
Demographic data				
Breed (*n* = 36)				
Spanish Pure breed		10 (33.3)		2 (25.0)
Lusitano		2 (6.7)		1 (12.5)
Hot-blooded		4 (13.3)		2 (25.0)
Warm-blood		4 (13.3)		0 (0.0)
Crossbred		7 (23.3)		3 (37.5)
Pony		1 (3.3)		0 (0.0)
Donkey		1 (3.3)		0 (0.0)
Sex (*n* = 38)				
Male		18 (60.0)		2 (25.0)
Female		6 (20.0)		6 (75.0)
Geldings		6 (20.0)		0 (0.0)
Age (year old)	7 (5/11.5)		6.5 (5/9.8)	
Clinical data				
T° (°C) (*n* = 37)	37.7 ± 0.7		38.5 ± 0.5	
HR (ppm) (*n* = 38)	50 ± 14		92 ± 17	
RR (bpm) (*n* = 38)	16 (12/24)		40 (24/43)	40 (24/43)
Mucosal membrane (*n* = 38)				
Pink		20 (66.3)		0 (0.0)
Pale		3 (10.0)		1 (12.5)
Congestive		6 (20.0)		6 (75.0)
Cyanotic		0 (0.00)		1 (12.5)
WBC (cells × 10^3^/µL)	7.350 ± 3.396		8.695 ± 3.704	
Lesion location (*n* = 39)				
Small intestine		9 (30.0)		3 (37.5)
Large intestine		16 (53.3)		4 (50.0)
Both intestines		3 (10.0)		0 (0.0)
Stomach		1 (3.3)		1 (12.5)
Uterus		1 (3.3)		0 (0.0)
Lesion type (*n* = 39)				
Non-strangulated		28 (93.9)		4 (50.0)
Strangulated/ruptured		2 (6.7)		4 (50.0)

Quantitative normally distributed data are expressed as mean ± SD or median (25th/75th) and categorical variables as N and per cent within the group. SD, standard deviation; n, population number; yo, years old; HR, heart rate; bpm, pulses per minute; RR, respiratory rate; bpm, breaths per minute; WCB, white blood cell count; EC, eosinophils cell count.

## Data Availability

The data presented in this study are contained within the article.

## Supplementary Materials

The following supporting information can be downloaded at: https://www.mdpi.com/article/10.3390/ani14010164/s1, Table S1: Individual demographic and clinical variables of 39 horses admitted to an equine hospital with acute abdominal pain.
