# Supplementary material for: Association between Eosinophil Count and Cortisol Concentrations in Equids Admitted in the Emergency Unit with Abdominal Pain
Source: Animals (Basel). 2024 Jan 4;14(1):164. doi: 10.3390/ani14010164 (PMC10778409; doi:10.3390/ani14010164)
Supplement: Supplementary file 1 [file animals-14-00164-s001.zip › animals-2740521-supplementary.pdf]

Table S1. Individual demographic and clinical variables of 39 horses admitted to an equine hospital with acute abdominal pain.

Breed	Sex	Age (yo)	Tª (ºC)	HR (ppm)	RR (bpm)	Mucosal membrane	Salivary cortisol (µg/dL)	WBC (× 10^9/L)	EC (× 10^9/L)	Diagnoses	Survival
Crossbreed	M	5	37.3	32	12	Pales	0.94	5.73	0.00	Small intestine enteritis and large colon impaction	Yes
Crossbreed	F	5	38.6	100	44	Pales	2.90	13.71	0.00	Large colon displacement	No
Spanish Pure Breed	M	3	38.4	80	24	Pink	0.99	7.92	0.02	Pelvic flexure impaction	Yes
Spanish Pure Breed	M	18	38.7	40	16	Pales	0.27	7.36	0.23	Spasmodic colic	Yes
Spanish Pure Breed	M	5	38.6	56	12	Pink	0.46	7.34	0.31	Large colon displacement	Yes
Spanish Pure Breed	M	12	39	50	20	Pink	0.52	18.66	0.13	Large colon displacement and small colon impaction	Yes
Spanish Pure Breed	F	5	37.5	40	12	Pink	0.65	12.10	0.24	Spasmodic colic	Yes
Spanish Pure Breed	F	8	39	100	40	Cyanotic	1.30	8.91	0.00	Large colon displacement	No
Crossbreed	F	7	38.1	90	40	Pink	0.30	9.44	0.18	Large colon displacement	Yes

Crossbreed	C		37.5	44	16	Pink	0.76	6.60	0.05	Large colon displacement and pelvic flexure impaction	Yes
Pony	C	7	37	44	12	Pales	0.96	7.21	0.06	Large colon displacement	Yes
Spanish Pure Breed	M	8	37.4	44	16	Pink	1.11	6.28	0.01	Pelvic flexure impaction	Yes
Spanish Pure Breed	M	3	37.7	44	16	Pink	1.12	9.36	0.04	Large colon displacement and small colon impaction	Yes
Crossbreed	C	14	37.8	44	12	Pink	0.59	4.49	0.11	Pelvic flexure impaction	Yes
Spanish Pure Breed	M	6	38.2	64	12	Congested	0.67	3.61	0.00	Duodenitis and proximal jejunitis	Yes
Warm blood	C	16	37.7	39	16	Pink	19.50	8.12	0.04	Large colon displacement	Yes
Warm blood	C	12	37.6	48	14	Pink	3.05	4.02	0.01	Large colon displacement and pelvic flexure impaction	Yes
Hot-blooded	F	5	37.7	100	40	Congested	2.00	7.15	0.00	Small intestinal volvulus	No
Warm blood	F	7	35.5	44	12	Pink	0.81	9.39	0.01	Duodenitis and proximal jejunitis	Yes

Spanish Pure Breed	M	5	39.1	76	32	Congested	0.77	7.30		Colithis	Yes
Lusitano	M	9	37.4	44	20	Pink	0.61	9.80	0.30	Gastric ulcers	Yes
Donkey	M	8	37	48	24	Pink	0.35	8.97	0.01	Large colon impaction	Yes
Crossbreed	M	2	38.1	52	20	Congested	0.51	20.50		Small intestinal volvulus	No
Lusitano	M	16	37	60	24		1.02	10.28	0.19	Sand	Yes
Hot-blooded	M	3	38	52	16	Congested	1.35	8.68	0.11	Duodenitis and proximal jejunitis	Yes
Hot-blooded	M	8	37.2	36	16	Pink	1.08			Duodenitis and proximal jejunitis and gastric ulcers	Yes
Spanish Pure Breed	M	6	37.7	40	16	Congested	2.12	3.00	0.02	Inguinal hernia	Yes
Hot-blooded	F	9	37.9	48	12	Pink	1.64	7.01	0.00	Uterine torsion	Yes
Crossbreed	M	6	37.9	40	16	Congested	0.38	6.74	0.30	Large colon impaction	Yes
Spanish Pure Breed	M	9		100	24	Congested	2.01	4.30		Gastric rupture	No

Warm-blood	M	5	37.8	44	16	Pink	0.27	2.70	0.01	Large colon displacement	Yes
LuYestaNo	F	9	37	84	70	Congested	0.45			Small intestinal volvulus	No
Crossbreed	F	10	37.9	96	24	Congested	1.64	5.00		Fecalith	No
Hot-blooded	F	10	37.6	36	12	Pink	1.25	4.87	0.03	Small intestine enteritis	Yes
Hot-blooded	F	10	38.5	100	24	Congested	1.83	5.01	0.01	Colithis	No
Spanish Pure Breed	M	18					0.54			Large colon impaction	
Crossbreed	F	18	36.9	60	60	Congested	0.91	6.50		Duodenitis and proximal jejunitis	Yes
	M		39.2	76	29	Pink	0.52	9.90	0.10	Small intestine enteritis	Yes
Crossbreed	C	10	37.8	60	26	Pink	2.26			Duodenitis and proximal jejunitis	Yes

yo, years-old; HR, heart rate; bpm, pulses per minute; RR, respiratory rate; bpm, breaths per minute; WCB, white blood cell count; EC, eosinophils count.
